# Grapevine Endophyte Endornavirus and Two New Endornaviruses Found Associated with Grapevines (*Vitis vinifera* L.) in Idaho, USA

**DOI:** 10.3390/v15061347

**Published:** 2023-06-10

**Authors:** Jennifer Dahan, Gardenia E. Orellana, Jungmin Lee, Alexander V. Karasev

**Affiliations:** 1 Department of Entomology, Plant Pathology and Nematology, University of Idaho, Moscow, ID 83844, USA; jdahan@uidaho.edu (J.D.); gardeniao@uidaho.edu (G.E.O.); 2 Horticultural Crops Production and Genetic Improvement Research Unit, Agricultural Research Service, United States Department of Agriculture, Corvallis, OR 97330, USA; jungmin.lee@usda.gov

**Keywords:** grapevine endophyte endornavirus, grapevine endornaviruses 1 and 2, Chardonnay, Cabernet franc

## Abstract

Five virus genomes, ranging between 12.0 and 12.3 kb in length and identified as endornaviruses, were discovered through a high-throughput sequencing (HTS) analysis of the total RNA samples extracted from two wine grape cultivars collected in the State of Idaho. One was found in a declining Chardonnay vine and was determined to be a local isolate of grapevine endophyte endornavirus (GEEV), and four others represented two novel endornaviruses named grapevine endornavirus 1 (GEV1) and grapevine endornavirus 2 (GEV2). All three virus genomes span a large, single open reading frame encoding polyproteins with easily identifiable helicase (HEL) and RNA-dependent RNA polymerase (RdRP) domains, while the GEV2 polyprotein also contains a glycosyltransferase domain. The GEV1 genome found in an asymptomatic Cabernet franc vine was related to, but distinct from, GEEV: the 5′-proximal, 4.7 kb segment of the GEV1 genome had a 72% identical nucleotide sequence to that of GEEV, while the rest of the genome displayed no significant similarity to the GEEV nucleotide sequence. Nevertheless, the amino acid sequence of the RdRP domain of GEV1 exhibited the closest affinity to the RdRP of GEEV. GEV2 was found in declining Chardonnay and asymptomatic Cabernet franc vines as three genetic variants exhibiting a 91.9–99.8% nucleotide sequence identity among each other; its RdRP had the closest affinity to the Shahe endorna-like virus 1 found in termites. In phylogenetic analyses, the RdRP and HEL domains of the GEV1 and GEV2 polyproteins were placed in two separate clades inside the large lineage of alphaendornaviruses, showing an affinity to GEEV and *Phaseolus vulgaris* endornavirus 1, respectively.

## 1. Introduction

Endornaviruses are a family of single-stranded (ss), positive-sense RNA viruses with genomes ranging between 9700 and 21,770 nucleotides (nt) long [1,2,3,4,5]. Viruses from the family *Endornaviridae* have been found in plants, insects, fungi, and oomycetes, where most of them are asymptomatic and accumulate large amounts of double-stranded (ds) RNA-replicative intermediates [1,2,3,5,6]. Phylogenetically, endornaviruses belong to a lineage of ssRNA viruses from the alphavirus-like supergroup and are placed as a sister clade to toga-, virga-, and closteroviruses [3,7]. Currently, the family *Endornaviridae* is comprised of two genera: *Alphaendornavirus*, which includes viruses found in plants, insects, fungi, and oomycetes, and *Betaendornavirus*, which includes fungal viruses [3,4]. All endornavirus genomes characterized so far do not encode genes for structural proteins and are, thus, assumed to be unable to establish a productive infection in their hosts, and also unable to be horizontally transmitted [2]. Vertical transmission is presumed to be the only means for the spread of endornaviruses in plants, while in fungal and oomycete hosts, horizontal transmission through hyphal anastomosis does occur, in addition to vertical transmission via spores [3,8].

Grapevines (*Vitis vinifera* L.) are woody perennials that have been cultivated vegetatively for at least 10,000 years [9], and hence, they are prone to the accumulation and spread of multiple virus species, some of which may not yet be known. More than 80 different viruses may be found infecting grapevines, and some 30 virus-related grapevine diseases and abnormalities are known [10,11]. A single endornavirus was identified in grapevine tissue from cv. Shiraz in South Africa, which is known as the grapevine endophyte endornavirus (GEEV) [12]. The host specificity of the virus is not exactly clear, since GEEV was originally found in grapevine tissue where no trace of a fungal host could be detected, but was also found in two endophytic fungal isolates from two different vines [12]. The presence of GEEV has not been reported outside of South Africa; no other endornaviruses have been reported to infect grapevines or grapevine-associated organisms up to now.

In 2021, there were approximately 526 ha of grapevine plantings in the state of Idaho, which resulted in 2100 tons of wine grapes produced. The Idaho wine industry is considered new and growing compared to the neighboring states of Washington and Oregon, with two main American viticulture areas (AVAs) established. Viruses are a concern for grape production in the state, with grapevine leafroll-associated virus 3 (GLRaV-3), grapevine fleck virus (GFkV), grapevine red blotch virus (GRBV), grapevine rupestris vein feathering virus (GRVFV), grapevine-associated tymo-like virus (GaTLV), and grapevine rupestris stem pitting-associated virus (GRSPaV) reported in the state over the years [13,14,15,16,17,18,19]. Some of these virus infections negatively impact grape quality [20,21]. The application of high-throughput sequencing (HTS) methods greatly facilitates the detection, identification, and discovery of new viruses in grapevines, expanding the number of viruses known to infect *V. vinifera* [11,22] and the number known in new geographic areas, including many viruses not previously reported in Idaho [15,16,17,18,19].

Here, we report the discovery of three endornaviruses in Idaho grapevines, including an Idaho isolate of GEEV and two new endornaviruses associated with several grapevine cultivars. All of these endornaviruses were discovered through the application of HTS and the subsequent validation and confirmation of virus presence by conventional RT-PCR and Sanger sequencing.

## 2. Materials and Methods

### 2.1. Grapevine Sampling and Sample Processing

Grapevine leaf and petiole samples were collected from two commercially operated vineyards in the Canyon (vineyard A) and Nez Perce (vineyard B) counties of Idaho. These two vineyards are about 420 km apart, situated in two different AVAs, and have different owners. A 38-year-old declining “Chardonnay” block was sampled in vineyard A in September 2020, and a 20-year-old healthy-looking “Cabernet franc” block was sampled in vineyard B in October 2020. Two samples from vineyard A, RB09 and RB12, and one from vineyard B, CC06, were submitted to an HTS analysis; an additional four samples from vineyard B and nine samples from four additional vineyards in Canyon County were collected in 2020 and subjected to RT-PCR testing. Vineyard A was also sampled in September 2021, along with two additional vineyards in Canyon County sampled in 2020. The sampling methodology followed a previously described protocol [15]. Briefly, four fully expanded leaves with complete petioles were collected per vine, from all sides of the canopy, and placed into a single plastic resealable bag labeled with the vine number and the name of the vineyard. These leaf samples were kept in a cooler with ice for 2–3 days until reaching the laboratory and kept in a cold room (4 °C) until final processing, which occurred 3–14 days after leaf collection. The petioles were cut off from the leaves and used for a subsequent extraction and analysis.

### 2.2. RNA Extraction and HTS Analysis

The plant tissue was ground in plastic meshed bags (Bioreba AG, Reinach, Switzerland), the total RNA was extracted using a Spectrum Plant Total RNA Kit (Sigma-Aldrich, St. Louis, MO, USA), and, following a ribodepletion using a RiboMinus Plant Kit for RNA-Seq (Invitrogen, Waltham, MA, USA), libraries were prepared using a Kapa RNA Hyper-Prep Kit (Roche, Indianapolis, IN, USA) with NEXTflex-Unique-Dual-Index-Barcodes-Set-C (BioO Scientific, Austin, TX, USA). After a bead-based size selection, the resulting libraries were multiplexed and subjected to Illumina high-throughput sequencing on a NovaSeq 6000 platform through the University of Idaho Genomics and Bioinformatics core facility. Between 30.2 and 63.0 million 250 bp paired-end reads per sample were produced. The raw reads were adapter- and quality-cleaned using Trimmomatic v0.38 [23] and mapped against the *V. vinifera* L. reference genome using bowtie2 v2.4.4 in the local mode [15]; unmapped paired-end reads were subjected to assembly using SPAdes v3.15.3 in RNA mode and analyzed using the BLASTn and DIAMOND programs [24]. A search for conserved protein domains was conducted using the conserved domain database (CDD), available at the NCBI [25,26].

### 2.3. Nucleic Acid Extraction, RT-PCR Testing, and Sanger Sequencing

For samples collected in September–October 2020, the total RNA was extracted from grapevine leaf and petiole tissues that were ground in plastic meshed bags (Bioreba) by following the Spectrum Plant Total RNA Kit (Sigma-Aldrich) instructions. Reverse transcription was performed using 4.5 µL of the extracted RNA in a 25 µL reaction mixture that contained 5× first-strand buffer (Promega, Madison, WI, USA), 2.5 mM dNTP, 3 µM oligo dT + random hexamers, rRNasin ribonuclease inhibitor (Promega), and M-MLV reverse transcriptase (Promega). Before the reverse transcription reaction, the RNA template was incubated at 70 °C for 5 min, and then the reverse transcription mix was added. The profile used included an initial incubation at 25 °C for 10 min, 42 °C for 50 min, and reverse transcriptase deactivation at 70 °C for 15 min prior to PCR. All PCR reactions were accomplished using GreenTaq (GenScript, Piscataway, NJ, USA) in a 20 µL reaction mixture that contained 10x GreenTaq buffer, 2.5 mM dNTP, 5 µM each of the forward primer and reverse primer, GreenTaq, and 2 µL of cDNA template. The PCR profile consisted of denaturing at 94 °C for 2 min; 35 cycles of 94 °C for 30 s, 55–65 °C for 30 s (depending on the melting temperature of the primers used), and 72 °C for 1 to 2 min (depending on the fragment length amplified); and a final extension for 10 min at 72 °C. Sanger sequencing was performed on RT-PCR fragments amplified from the total RNA extracted from the infected grapevine plants as described above, and by following a previously described protocol [27]. The primers used to amplify these DNA fragments are listed in Supplemental Table S1. The PCR fragments were treated with ExosapIt (Affymetrix, Cleveland, OH, USA) and submitted for sequencing to Elim Biopharmaceuticals, Inc. (Hayward, CA, USA).

### 2.4. Sequence and Phylogenetic Analysis

The phylogenetic trees for the HEL and RdRP proteins were generated based on the set of sequences provided in a report by the International Committee on Taxonomy of Viruses (ICTV) on the family *Endornaviridae* [3,4], with the addition of GEV-1, GEV-2, and GEEV-Ch (this work) and the Geranium carolinianum endornavirus 1 sequences (GcEV1) [28]. The grapevine leafroll-associated virus 1 reference sequence was used as an outgroup. The sequences of the HEL and RdRP domains from each virus were aligned using MAFFT in the g-insi mode, as implemented in Geneious Prime 2023 (default parameters). A maximum likelihood tree was inferred in IQtree 2 [29,30] using ModelFinder for the best model selection [31] and the SH-alRT, abayes, and UFBoot tests for branch support estimation, available in IQtree 2 [32,33,34]. The outgroup was then forced at the root in FigTree v1.14.4.

## 3. Results

### 3.1. Endornavirus Sequences Revealed by HTS in Grapevine Leaf and Petiole Tissue

Five contigs, ranging between 12,030 and 12,279 nt in size and clearly related to endornaviruses, were revealed by HTS and the associated sequence analyses in three of the grapevine samples collected from two cultivars, Chardonnay and Cabernet franc, in vineyards A and B, respectively (Table 1).

All five contigs contained a single open frame that encoded large polyproteins spanning the HEL and RdRP domains identified by the CDD program searches, with the RdRP domain identified as an endornavirus-specific RNA-dependent RNA polymerase. One of these contigs, which was 12,279 nt long and found only in one Chardonnay sample, RB12, was 96.3% identical to GEEV at the nucleotide sequence level (Table 2) and was, thus, identified as a local isolate of the virus, named GEEV-Ch. Another, a 12,083 nt contig found in one Cabernet franc sample, CC06, exhibited a limited 72.4% identity to GEEV at the nucleotide level, but only in the 5′-terminal, 4700 nt fragment of the genome (Table 2), and no other hits were obtained in the BLASTn searches through the GenBank database; this contig was provisionally named grapevine endornavirus 1 (GEV1). Three other contigs, two from the same Cabernet franc sample, CC06, where GEV1 was found and the third from a Chardonnay sample, RB09, exhibited no hits in BLASTn searches through the GenBank database, but were found to exhibit 91.9–99.8% nucleotide sequence identity between each other in pair-wise comparisons (Table 2). These three sequences were, thus, assumed to represent three genetic variants of a novel endornavirus, which was named grapevine endornavirus 2 (GEV2) with designations for genetic variants based on the grapevine cultivar where they were found: GEV2-Ch, GEV2-Cf1, and GEV2-Cf2 (Table 1).

Three contigs, representing GEEV-Ch, GEV1, and GEV2-Cf2, were assembled in the stranded library as positive-sense genomes, and two contigs, GEV2-Ch and GEV2-Cf1, were assembled as negative genomes. The reasons for these strand polarity differences were not clear; perhaps the relative levels of (+) and (-) virus strands varied between the samples at the time of collection. To stay consistent, all genome sequences were deposited in GenBank in the positive sense. To confirm the presence of GEEV-Ch, GEV1, and GEV2 in the original samples and validate the sequence determined by HTS, RT-PCR was conducted with the specific primers listed in Table S1. The presence of all virus isolates in the original Chardonnay and Cabernet franc samples was confirmed for all five endornavirus sequences by the Sanger sequencing of each PCR product (see Table 1).

### 3.2. Genome Organization and Phylogeny of the New Grapevine Endornaviruses

**Figure 1:**
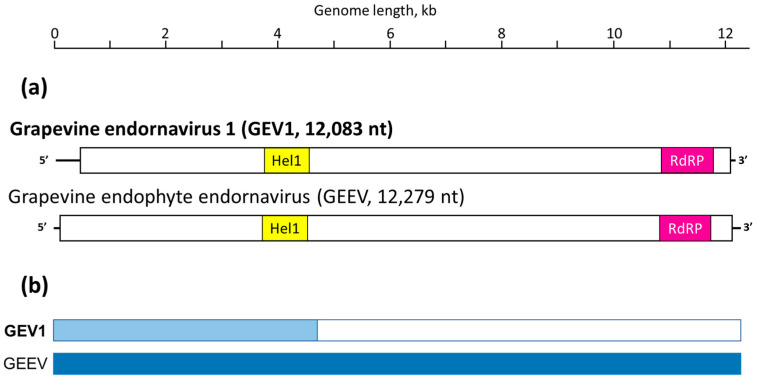
Schematic representation of the grapevine endornavirus 1 (GEV1) genome side-by-side with grapevine endophyte endornavirus (GEEV, JX678977). (**a**) GEV1 and GEEV genomes encode a single open reading frame, with conserved protein domains designated with like colors; Hel1 = helicase type 1, RdRP = RNA-dependent RNA polymerase. (**b**) Schematic diagram of a possible recombinant structure of GEV1: GEEV genome was arbitrarily set as a “parental” genome (blue color), with GEV1 genome containing a ca. 4.7 kb segment (light blue color) with 72% sequence identity to the “parental” GEEV genome at the nt level; the rest of the GEV1 genome, ca. 7.3 kb, displayed no significant similarity to GEEV at the nucleotide sequence level (white color).

**Figure 2:**
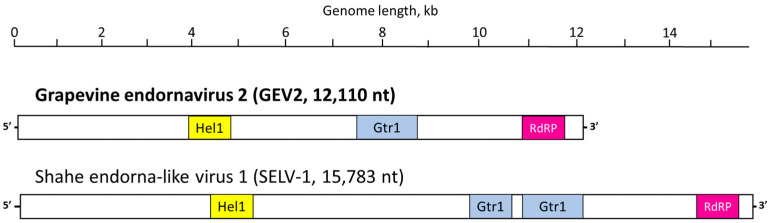
Schematic representation of the grapevine endornavirus 2 (GEV2) genome side-by-side with Shahe endorna-like virus 1 (SELV-1, KX883795); virus genomes are visualized as positive RNA strands. A single open reading frame is represented as a rectangle, with conserved protein domains designated with like colors; Hel1 = helicase type 1, Gtr1 = glycosyltransferase type 1, RdRP = RNA-dependent RNA polymerase.

The 12,279 nt contig identified as a local Idaho isolate of GEEV (GEEV-Ch, Figure 1a) spanned a single ORF, potentially encoding a 4029 aa protein product (nt positions 5–12,094). The 12,083 nt contig was identified as a new virus, and named GEV1 (Figure 1a), spanning a single ORF capable of coding for a 3842 aa protein product (nts 522–12,050). These hypothetical polyproteins for both viruses, GEEV-Ch and GEV1, contained easily identifiable conserved domains for HEL and RdRP located in very similar positions (Figure 1a). The three 12,030-to-12,032 nt contigs found in two grapevine samples, RB09 and CC06 (Table 1), and identified as three genetic variants of a new virus named GEV2, spanned a single ORF potentially encoding a 3992 aa protein product (nts 41–12,019). This polyprotein encoded by the GEV2 genome contained three identifiable conserved domains, i.e., HEL, glycosyltransferase, and RdRP (Figure 2).

**Figure 3:**
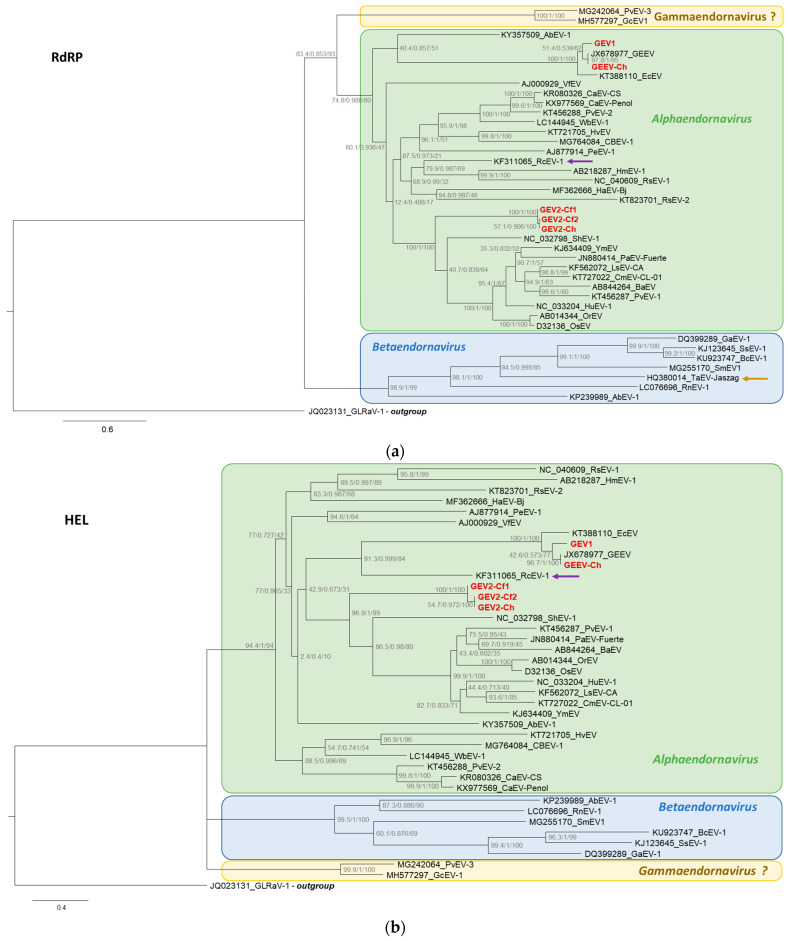
Phylogenies of endornavirus (**a**) RdRP and (**b**) HEL domains. Predicted polyproteins from GEV1, GEV2-Cf1, GEV2-Cf2, GEV2-Ch, and GEEV-Ch endornavirus genomes were added to the endornavirus RdRP and HEL alignments. A maximum-likelihood phylogenetic tree constructed in IQtree2, with ModelFinder for the best-fit model and branch support estimates with SH-aLRT/abayes/UFBoot (shown at nodes). Branch lengths are drawn to scale, with the scale bar showing the number of substitutions per site. The outgroup (GLRaV-1, a closterovirus) was forced at the root in FigTree v1.4.4.

All three endornaviruses found here to be associated with grapevines, including GEEV, were firmly placed in the Alphaendornavirus clade based on both the RdRP and HEL phylogenies (Figure 3a,b). In the RdRP phylogeny (Figure 3a), GEEV-Ch and GEV1 were placed in a distinct clade closely related to Erysiphe cichoracearum alphaendornavirus (EcEV) [35] from a fungal pathogen of wheat. In the HEL phylogeny (Figure 3b), GEEV and GEV1 were placed in the same clade with EcEV; however, in this phylogenetic tree, another virus, Rhizoctonia cerealis endornavirus 1 (RcEV-1) [36], was included as a sister clade in the same lineage. Interestingly, the RdRP of this RcEV-1 was placed in a lineage of alphaendornaviruses distant from the GEEV/GEV1 clade and close to Rhizoctonia solani endornavirus 1 (RsEV-1; Figure 3a). This may suggest a possible past recombination event in the RcEV-1 genome, bringing together the RdRP and HEL domains from parental genomes belonging to different alphaendornavirus clades. The three GEV2 sequences determined in this work were phylogenetically placed in a distinct and tight clade that was a sister clade to the Shahe endorna-like virus 1 (ShEV-1) found in a termite sample [6] and to a lineage of plant endornaviruses, including PvEV-1 (Figure 3a,b), for both the RdRP and HEL domains.

When comparing the RdRP and HEL phylogenies for alpha- and betaendornaviruses (Figure 3a,b), an additional example of a possible ancient recombination event in an endornavirus genome was noted. Specifically, a fungal virus from the summer truffle, Tuber aestivum endornavirus, isolate Jaszag (TaEV_J) [37], was placed among betaendornaviruses in the RdRP phylogeny (Figure 3a). Unexpectedly, its HEL domain could not be aligned reliably with the HEL domains of other alpha- and betaendornaviruses, resulting in an extremely long branch in a phylogenetic tree (not shown) that had to be removed to avoid interfering with the HEL phylogeny of endornaviruses (Figure 3b). Similar to the RcEV-1 genome, it is possible that the RdRP and HEL domains in the TaEV genome were brought together due to a recombination event between two parental genomes from two different genera of endornaviruses.

### 3.3. Prevalence of the Three Endornaviruses in Wine Grapes in Idaho

Of the 16 grapevine samples collected in September–October 2020 in six vineyards, three were found to contain endornaviruses by HTS (Table 1). One Chardonnay vine sample from vineyard A was GEEV-positive (sample RB12, Table 1), and another vine sample from the same Chardonnay block of vineyard A was GEV2-positive (sample RB09, Table 1). One Cabernet franc vine sample had three distinct endornavirus sequences, GEV1 and two genetic variants of GEV2 (sample CC06, Table 1). No endornavirus sequences were found in the other nine samples collected in 2020 in Canyon County from four additional vineyards, tested by RT-PCR. Eighteen additional grapevine samples collected in September 2021 from vineyard A and two additional vineyards in Canyon County were tested for the presence of GEEV, GEV1, and GEV2 using RT-PCR and specific primers. These 18 samples came from three vineyards located in Canyon County, Idaho, with five samples coming from the same vineyard A where the original RB09 and RB12 samples were taken in the fall of 2020 for the HTS analysis. Two out of the three Chardonnay samples collected in vineyard A in 2021 were found to be positive for the presence of GEV2, and more specifically, for the GEV2-Ch genetic variant. The identity of these two GEV2-Ch-specific PCR products was confirmed by Sanger sequencing. Unfortunately, the owner of vineyard A took out a significant section of the Chardonnay block in the spring of 2021 due to low productivity, and the same plants tested in 2020 could not be tested again. Three additional samples collected in vineyard B in 2020 from the cultivars Chardonnay, Negroamaro, and Riesling, besides the original CC06 from Cabernet franc, were found to be GEV1-positive by RT-PCR with specific primers (Table S1); the identity of these GEV1-specific PCR products was confirmed by Sanger sequencing. All four samples collected in 2020 from four grapevine cultivars in vineyard B exhibited no disease symptoms; hence, the presence of GEV1 was not linked to any apparent abnormality.

## 4. Discussion

The three viruses found to be associated with grapevines in Idaho, USA, expand the number of endornaviruses infecting grapevines to three: GEEV, GEV1, and GEV2 (Table 1). GEEV was previously reported from one red cultivar, Shiraz, in South Africa [12]. In Idaho, GEEV-Ch was found in a white cultivar (Chardonnay) displaying decline symptoms; the same plant was also infected with GLRaV-3, GRVFV, and GaTLV [17,18] and the role of GEEV-Ch, if any, in the induction of these decline symptoms remains unclear. GEV1 was found in the asymptomatic wine grapes of four cultivars, two red (Cabernet franc and Negroamaro) and two white (Chardonnay and Riesling), and was not linked to any visual disease symptoms. Three genetic variants of GEV2 were identified in two different cultivars, a white cultivar (Chardonnay) displaying symptoms of decline and an asymptomatic red cultivar (Cabernet franc)—in this latter case, the same Cabernet franc plant contained two genetic variants of GEV2 (Table 1). The GEV2-positive Chardonnay plant exhibiting decline symptoms had multiple other viruses present, GLRaV-3, GRVFV, and GaTLV [17,18], and the contribution of GEV2 to the decline in this 40-year-old Chardonnay block was unclear. Since all three of the identified viruses, GEEV, GEV1, and GEV2, belong to the family *Endornaviridae*, they may be persistent and asymptomatic in their grapevine host.

Endornaviruses comprise a family of persistent viruses that lost the ability to form virus particles and the ability to be transmitted horizontally, and became dependent on vertical transmission in their hosts [2,3]. The known endornaviruses have been found in a variety of hosts, including plants, fungi, and oomycetes [2]; genome sequences related to endornaviruses have been discovered in arthropods as well [38]. It is hypothesized that the reliance of endornaviruses on vertical transmission led to a symbiotic relationship with their hosts and, therefore, endornaviruses do not induce disease symptoms [1,2,3].

Endornaviruses have a linear RNA genome ranging between 10,000 and 17,000 nt that codes for a single polyprotein spanning the conserved HEL and RdRP domains and may also contain additional conserved domains, such as glycosyltransferase and protease domains [2,3]. A group of endornaviruses with a larger genome of up to 21,770 nt was described from fungal plant pathogens and coded for a second ORF downstream of the RdRP-encoding ORF1 [5,39]. Phylogenetically, the RdRP of endornaviruses is placed as a sister clade to a lineage that includes togaviruses, virgaviruses, negeviruses, bromoviruses, and closteroviruses, which are all within the large alphavirus-like supergroup that also includes the *Tymovirales* clade [7]. All three endornaviruses found here to be associated with grapevines, GEEV, GEV1, and GEV2, were firmly placed in the family *Endornaviridae* and, more specifically, in the clade of the genus *Alphaendornavirus* based on the RdRP phylogeny (Figure 3a). The three GEV2 sequences found to be associated with grapevines were placed in a tight lineage that is a sister clade to the Shahe endorna-like virus 1 found in a termite sample (Figure 3a). In our case, the leaf and petiole samples were used for the HTS analysis and RT-PCR tests, suggesting the association of the three found endornaviruses, GEEV, GEV1, and GEV2, with the plant (grapevine) host. The only grapevine-associated endornavirus known until now, GEEV, was reported to be found in both plant (grapevine) and fungal hosts [12], suggesting possible horizontal transmission between the two host types [12]. Here, GEV1 and GEV2 infections were not associated with any fungal infections and were, thus, assumed to have plant hosts.

In the current classification, the family *Endornaviridae* is divided into two genera, *Alphaendornavirus* and *Betaendornavirus*, based on the RdRP phylogenies and the genome domain architecture [3,4]. All five endornavirus sequences representing three virus species, GEEV, GEV1, and GEV2, that were found to be associated with wine grapes in Idaho, USA, were easily classified as alpaendornaviruses based on their RdRP and HEL phylogenies (Figure 3). The division between alpha- and betaendornaviruses based on RdRP phylogeny is clearly supported by the phylogeny of the HEL domain (Figure 3a,b). However, two of the viruses, Phaseolus vulgaris endornavirus 3 (PvEV-3) and Geranium carolinianum endornavirus 1 (GcEV-1), were placed together in a separate clade that may be viewed as a sister clade to the alphaendornavirus lineage for RdRP and a stand-alone clade for HEL phylogenetic trees (Figure 3a,b). Based on this placement in a separate, tight clade for the HEL and RdRP phylogenies, the similar domain architecture of PvEV-3 and GcEV-1, involving the presence of HEL, C97 peptidase, UGT, and RdRP domains, and an overall high amino acid identity level between each other [28,40], we propose to classify PvEV-3 and GcEV-1 into a distinct genus with the tentative name Gammaendornavirus (see Figure 3a,b).

The close RdRP phylogenetic placement has been noted for PvEV-3 and GcEV-1 before [5,28,41]. In 2021, Li et al. [5] presented a detailed phylogeny for the RdRP domains of endornaviruses and demonstrated a basal position of the PvEV-3/GcEV-1 clade to all other alphaendornavirus clades, albeit not proposing a new taxonomic unit. Our phylogeny for the HEL domain of endornaviruses (Figure 3b) complements the previously available phylogenetic trees for RdRP domains [5,28,41] and provides an additional argument for the creation of a third genus in the family *Endornaviridae*, which we propose to name the genus Gammaendornavirus. Incidentally, the name Gammaendornavirus was proposed for a phylogroup within the genus *Alphaendornavirus* earlier [42], but the proposal was based on an RdRP phylogeny only and for a relatively small number of endornavirus sequences available at the time; this proposal was later rejected [5]. It was put forward again recently [43] for an alleged lineage of endorna-like viruses based on RdRP phylogeny only; however, it was, in fact, applied to a lineage of virga- and negev-like viruses, which did not include PvEV-3 and GcEV-1, and actually included no endorna- or endorna-like viruses [43], apparently in error. Here, we propose the use of this name to designate a new lineage of endornaviruses, constituting a sister clade to alphaendornaviruses in the phylogenies of the RdRP and HEL domains and having a similar domain architecture in their polyproteins.

The persistent nature of endornaviruses in plants suggests the presence of the virus genome in all cells at a relatively low copy number due to a tight control of virus replication and the complete lack of horizontal transmission [2]. Both the lack of horizontal transmission and a low copy number of endornaviral RNA in plant cells may be used to argue against the recombination and high rate of the evolution characteristic of RNA viruses. Here, we present evidence of recombination involved in the evolution of endornaviruses. First, the genome of the newly found GEV1 is apparently built from two segments, with a 5′-terminal 4.7 kb segment coming from a parent closely related to GEEV (72% nt identity) and the remaining ca. 7.4 kb downstream segment coming from an alphaendornavirus only distantly related to GEEV (no significant nt similarity) (Figure 1b). The relationship of this 3′-terminal section of the GEV1 genome to GEEV can be detected at the amino acid sequence level (Figure 3a), but not at the nucleotide sequence level. Second, two additional examples of possible recombination events involved in the endornavirus evolution are the RcEV-1 and TaEV-J genomes, where the RdRP and HEL phylogenetic placements differed between the RdRP and HEL phylogenetic trees (Figure 3a,b). Both lines of evidence may be viewed as suggesting the existence of horizontal transmission of some sort for endornaviruses that might bring two different endornavirus genomes into the same cell and facilitate exchanges of different conserved domains between them. One hypothesis explaining these recombination events visible in the GEV1, RcEV-1, and TaEV-J genomes may be the possibility of such a recombination event occurring in fungal and oomycete hosts, where horizontal transmission through hyphal anastomosis has been demonstrated [3,4,8]. For GEV1, this hypothesis may mean that the virus can perhaps “shuttle” between plant (grapevine) and fungal hosts, similar to GEEV [12].

## 5. Conclusions

Three endornaviruses were discovered in Idaho grapevines, including an Idaho isolate of GEEV, which represents the first finding of this virus outside of South Africa. Two new endornaviruses, GEV1 and GEV2, were found to be associated with two grapevine cultivars from two different vineyards and from two separate AVAs in Idaho. All of these endornaviruses were discovered through the application of HTS and the subsequent validation and confirmation of virus presence using conventional RT-PCR and Sanger sequencing. A phylogenetic analysis of the HEL and RdRP domains of GEEV, GEV1, and GEV2 placed all three as species of the genus *Alphaendornavirus*, family *Endornaviridae*. The phylogenies of these replication-associated domains suggest the separation of two endornaviruses, PvEV-3 and GcEV-1, into a new genus with the provisional name Gammaendornavirus. The data obtained indicate that recombination is involved in the evolution of endornaviruses. 

## Figures and Tables

**Table 1:** Summary of endornavirus sequences from three grapevine samples collected in 2020 revealed through the use of high-throughput sequencing (HTS) and RT-PCR.

Virus Isolate Name ^1^	Contig Size, nt	Number of HTS Reads Mapped	Polyprotein Size, aa	Virus ID ^2^	GenBank Accession	RT-PCR Validation ^3^	Vineyard	Samples with This Virus
GEEV-Ch	12,279	1478	4029	GEEV	OR075262	+	A	RB12
GEV1	12,083	608	3842	New	OR075263	+	B	CC06
GEV2-Ch	12,031	2097	3992	New	OR075264	+	A	RB09
GEV2-Cf1	12,030	1100	3992	New	OR075265	+	B	CC06
GEV2-Cf2	12,030	2484	3992	New	OR075266	+	B	CC06

^1^ GEEV-Ch = grapevine endophyte endornavirus, Chardonnay isolate; GEV1 = grapevine endornavirus 1; GEV2-Ch = grapevine endornavirus 2, Chardonnay isolate; GEV2-Cf1 = grapevine endornavirus 2, Cabernet franc isolate 1; GEV2-Cf2 = grapevine endornavirus 2, Cabernet franc isolate 2. ^2^ GEEV = grapevine endophyte endornavirus; “New” = newly discovered endornavirus. ^3^ Total RNA was extracted from leaf and petiole samples as described in the Materials and Methods, followed by RT-PCR and subsequent Sanger sequencing of the PCR product.

**Table 2:** Pair-wise comparisons between the grapevine endornavirus whole genome sequences (bottom left section) and whole polyprotein amino acid sequences (top right section). Percent numbers show the identity level, with the coverage in brackets; “-”, no significant similarity.

	GEEV ^1^	GEEV-Ch	GEV1	GEV2-Ch	GEV2-Cf1	GEV2-Cf2
**GEEV ^1^**		**98.7%** (100%)	**73.2%** (95%)	**33.5%** (18%)	**33.7%** (20%)	**33.5%** (18%)
**GEEV-Ch**	**96.3%** (99%)		**73.0%** (95%)	**33.5%** (18%)	**33.7%** (20%)	**33.5%** (18%)
**GEV1**	**72.4%** (38%)	**72.3%** (38%)		**31.5%** (20%)	**26.4%** (29%)	**31.5%** (19%)
**GEV2-Ch**	-	-	-		**96.6%** (100%)	**100%** (100%)
**GEV2-Cf1**	-	-	-	**92.0%** (99%)		**96.6%** (100%)
**GEV2-Cf2**	-	-	-	**99.8%** (99%)	**91.9%** (99%)	

^1^ GEEV = grapevine endophyte endornavirus, Shiraz isolate, GenBank accession number: JX678977. Other abbreviations are: GEEV-Ch = GEEV, Chardonnay isolate; GEV1 = grapevine endornavirus 1; GEV2-Ch = grapevine endornavirus 2, Chardonnay isolate; GEV2-Cf1 = GEV2, Cabernet franc isolate 1; GEV2-Cf2 = GEV2, Cabernet franc isolate 2.

## Data Availability

All data are available upon reasonable request.

## Supplementary Materials

The following supporting information can be downloaded at: https://www.mdpi.com/article/10.3390/v15061347/s1, Table S1: Summary of primers used in this study and their sequences.
